# Anæsthesia in Puerperal Convulsions

**Published:** 1862-04

**Authors:** P. S. Dorland

**Affiliations:** East Hamburgh, N. Y.


ART. III.—*Anæsthesia in Puerperal Convulsions—By* P. S. DORLAND M. D., *East Hamburgh, N. Y.*

(Communicated to the Buffalo Medcal and Surgical Journal.)

Although the New York Academy of Medicine may have exhausted the subject of Anæsthesia in labor, yet perhaps a little more evidence in regard to the "Agent" in Puerperal Convulsions might be of service, and I therefore intrude upon your notice the report of a singular case.

On the 23d of October, 1861, I was summoned to visit Mrs. C., who the messenger stated, was in a fit. I arrived at 11 P. M; found patient quiet, but totally insensible. She is of a strong constitution, short stature, nervo-sanguine temperament, American, 35 years of age, and had completed the seventh month of her fourth pregnancy. Was taken ill at 9 o'clock, and in two hours had four violent convulsions. I directly noticed a drawing of the mouth to one side, with twitching of the facial muscles, eyes turned obliquely upward, a tremulous winking motion of lids, arms beginning to draw up, and one hand clenched in the hair, tearing out a large handful as the convulsions increased, the tongue protruding to one side, with bloody mucus escaping from the mouth, respiration very irregular and spasmodic. After the convulsion had entirely subsided, respiration became regular and slightly stertorous, pulse 108, soft and compressible, skin moist, head hot, and os uteri not at all dilated.

Under these circumstances I resorted to anæsthesia to control the convulsions, using for that purpose a mixture of chloroform and sulphuric ether, equal parts. I endeavored to keep up partial anæsthesia continually, but gave "the mixture" more freely at the slight twitching of the muscles that preceded each pain. In this way the interval of convulsions was lengthened from thirty minutes to two hours, as the first effect of the anæsthetic. The next convulsion was materially lessened in severity, and after that the interval extended to 2½ and 3 hours, nevertheless the pains increased gradually in frequency and were very strong. At 12 M. of 24th, just 12 hours after commencing exhibition of chloroform, os uteri was dilated to size of a half dollar, but very firm and unyielding, soft parts not relaxed, head can be easily detected through the membranes. But little progress having been made for three hours, although pains were occurring every five minutes, and forcible, we thought best to try the lancet, to produce if possible, more perfect relaxation. With Dr. Jarvis in counsel, patient's head and shoulders were slightly elevated, vein opened, and blood flowed freely of *bright arterial color*. No more than 10℥ were taken before a weak, sighing, respiration, extreme pallor, and a pulse 120, irregular and thready, warned us to desist. A violent convulsion occurred ten minutes after. Her chances now seemed to us very small. We immediately ruptured the membranes, and gave the anæsthetic very freely. Pains continued good, but labor progressed very slowly. All the assistance, manually that could be given, was used. For two hours after the bleeding, but little change occurred, then relaxation occurred more rapidly. One more convulsion occurred at 2½ o'clock and at 3 P. M. she was delivered of a female child weighing from 4 to 5 lbs., which lived 12 hours. No anæsthetic was used after delivery, but a full dose of morphine given. Two convulsions occurred after, and then patient sank down very weak and prostrate. Stimulants and anodynes were now used freely to rally the depressed nervous system. Pulse soon became stronger, but insensibility remained till 12 M. of 25th, nearly forty hours. The next forty-eight hours she was wakeful and restless, then made a rapid recovery. Before consciousness returned hyd. sub. mur. 12 grs. was given in three doses, 4 grs. each, followed by oleum ricini, and a good operation followed. In two weeks the woman was doing her work.

Four years ago this same patient, while in labor, was attacked the same way; was insensible over 40 hours, had 22 convulsions, was bled over 40 ounces, and as a last resort chloroform was used. She lived, but was an invalid for months, being weak and nervous. She was attended at that time by Drs. Emmons, House, and Davis; from the latter I obtained these particulars. In her last attack the number of convulsions was sixteen; the amount of anæsthetic used was nearly one pound, on a large handkerchief.

*Query*—Does puerperal convulsions depend mainly on cerebral congestion? or an intense cerebro-spinal irritation?

